# Evidence for Teaching in an Australian Songbird

**DOI:** 10.3389/fpsyg.2021.593532

**Published:** 2021-02-22

**Authors:** Hollis Taylor

**Affiliations:** Macquarie School of Social Sciences, Macquarie University, Sydney, NSW, Australia

**Keywords:** vocal learning, culture, teaching, tutoring, pied butcherbird, birdsong

## Abstract

Song in oscine birds (as in human speech and song) relies upon the rare capacity of vocal learning. Transmission can be vertical, horizontal, or oblique. As a rule, memorization and production by a naïve bird are not simultaneous: the long-term storage of song phrases precedes their first vocal rehearsal by months. While a wealth of detail regarding songbird enculturation has been uncovered by focusing on the apprentice, whether observational learning can fully account for the ontogeny of birdsong, or whether there could also be an element of active teaching involved, has remained an open question. Given the paucity of knowledge on animal cultures, I argue for the utility of an inclusive definition of teaching that encourages data be collected across a wide range of taxa. Borrowing insights from musicology, I introduce the Australian pied butcherbird (*Cracticus nigrogularis*) into the debate surrounding mechanisms of cultural transmission. I probe the relevance and utility of mentalistic, culture-based, and functionalist approaches to teaching in this species. Sonographic analysis of birdsong recordings and observational data (including photographs) of pied butcherbird behavior at one field site provide evidence that I assess based on criteria laid down by Caro and Hauser, along with later refinements to their functionalist definition. The candidate case of teaching reviewed here adds to a limited but growing body of reports supporting the notion that teaching may be more widespread than is currently realized. Nonetheless, I describe the challenges of confirming that learning has occurred in songbird pupils, given the delay between vocal instruction and production, as well as the low status accorded to anecdote and other observational evidence commonly mustered in instances of purported teaching. As a corrective, I press for an emphasis on biodiversity that will guide the study of teaching beyond human accounts and intractable discipline-specific burdens of proof.

## Vocal Learning

Song in oscine birds relies upon the learned acquisition of heard models (Koehler, 1951; Thorpe, 1951a,b; Baptista and King, 1980; Kroodsma and Baylis, 1982; Baker and Cunningham, 1985; Catchpole and Slater, 1995). Vocal learning allows for diversity and complexity not possible in innate song (Slater, 1986; Beecher and Brenowitz, 2005). The plasticity provided by the learning process may also give rise to variations that enable individual or kin recognition and other essential communication (Miller, 1979; Feekes, 1982). Vocal behavior, including acoustic features and the amount of learning incorporated in them, differs widely across songbird species (Devoogd, 2004). For instance, the white-crowned sparrow *Zonotrichia leucophrys* must only master a single stereotypical phrase (Marler and Tamura, 1964), while the improbably rich repertoire of the brown thrasher *Toxostoma rufum* runs to thousands of phrases, some likely improvised (Kroodsma and Parker, 1977).

Vocal learning can be vertical (from parents), horizontal (from peers), or oblique (from unrelated birds) (Marler and Tamura, 1964; Lynch et al., 1989; Baptista and Gaunt, 1997). Although the processes that guide song development can overlap (Nottebohm, 1969) and the time span varies widely, song acquisition by and large transpires in the first year of life during a critical sensitive period (Marler, 1970a; Nelson et al., 1995). Striking parallels exist between human speech and birdsong in their developmental stages (Darwin, 1871/1981; Marler, 1970b; Doupe and Kuhl, 1999; Hauser et al., 2002; Goldstein et al., 2003; Jarvis, 2004; Fitch, 2005; Merker and Okanoya, 2007; Bolhuis et al., 2010; Bolhuis and Everaert, 2016; Aamodt et al., 2020). Some songbirds retain vocal plasticity into adulthood; these open-ended learners may routinely learn new repertoire, re-open seasonally, or deliver previously unused song phrases (Yasukawa et al., 1980; Marler, 1981, 1990; Nottebohm et al., 1986; Brown et al., 1988; McGregor and Krebs, 1989; Nottebohm, 1989, 1993; Trainer, 1989; Adret-Hausberger et al., 1990; Chaiken et al., 1994; Mountjoy and Lemon, 1995; Payne, 1996; Baptista and Gaunt, 1997; Doupe and Kuhl, 1999; Adret, 2004; Taylor, 2017).

Young birds can commit songs to memory with very limited exposure (Hultsch and Todt, 1989b; Böhner, 1990; Peters et al., 1992). The “sensory” (memorization) and “motor” (sound reproduction or “sensorimotor”) phases of song learning are not typically simultaneous (Thorpe, 1961; Todt et al., 1979; Kroodsma and Pickert, 1984; Petrinovich, 1985; Moorman and Bolhuis, 2016; but see Tchernichovski et al., 2001; Roper and Zann, 2006). First attempts at production may begin weeks or months after the receipt and storage of song (Marler and Peters, 1982a; Marler, 1997; Podos et al., 2009). The separation between auditory memory formation and vocal production is common not just in songbirds but also extends to parrots (Pepperberg, 1997) and dolphins (Kremers et al., 2011). The stored neural representation of a tutor’s song may be activated and mediated through mirror neurons (Prather et al., 2008; Tchernichovski and Wallman, 2008; Mooney, 2014), whereby birds are able to compare and shape subsequent vocal rehearsals to their memory.

Since humans are the only primate species with the specialized cerebral capacity for vocal learning, this rare trait makes songbirds a powerful and versatile model for research on an array of problems. Intelligent, open to experimental manipulation, and inexpensive to feed and house, birds are easier to study and document than many species, although the quest for experimental control in laboratory experiments risks failing to give a complete description of real-world tutor/tutee relationships. Countless “professional” songbirds have been conscripted into ethology and neurobiology research projects, but despite the vast amount of scientific attention paid to vocal learning, it is striking that “tutor” may refer to a tape recorder or a human stand-in, while the term “live model” may stand in for “teacher” (e.g., Kroodsma and Pickert, 1984).

Efforts of everyday people to encourage songbirds’ vocal learning indicate an appreciation of this avian capacity pre-dating scholarly papers on the subject. Song tutoring of caged birds via mechanical instruments like the *serinette* (in the first half of the 18th century) was a popular hobby, with roots extending back to at least the third century BCE (Ord-Hume, 2001). The earliest print manuals for training songbirds on a flageolet, recorder, or flute date from *c*1700; *The Bird Fancyer’s Delight* targets nightingales *Luscinia megarhynchos*, canaries *Serinus canaria domestica*, blackbirds *Agelaius phoeniceus*, bullfinches *Pyrrhula pyrrhula*, starlings *Sturnus vulgaris*, and other songbirds, and is still in print (Godman, 1955/1717).

The learning trajectories of oscines are remarkably diverse (Beecher and Brenowitz, 2005). Birds raised in artificial conditions without access to song models produce “isolate,” or untutored, song, much of which develops abnormally (Marler et al., 1972; Nottebohm, 1972; Williams et al., 1993; Catchpole and Slater, 1995; Kroodsma and Miller, 1996; Marler, 1997; Payne and Payne, 1997; Doupe and Kuhl, 1999). Isolation as an acceptable and adequate context for the study of vocal communication, however, has not gone without critique (West et al., 1997). With the advent of magnetic tape recordings, researchers began to present conspecific and even allospecific songs to naïve, acoustic isolates. At one end of the continuum are species who learn to vocalize good copies of tape models played back on a loudspeaker (e.g., chaffinch *Fringilla coelebs*, Thorpe, 1958; e.g., song sparrow *Melospiza melodia*, Peters et al., 1992; e.g., swamp sparrow *Melospiza georgiana*, Marler and Peters, 1982b; e.g., white-crowned sparrow *Zonotrichia leucophrys*, Konishi, 1965; Marler, 1970a; Baptista and Petrinovich, 1986). For some species, social and/or visual contact with a tutor improves both quantity and quality of song acquisition (e.g., cardinal *Richmondena cardinalis*, Dittus and Lemon, 1969; e.g., canary, Waser and Marler, 1977; e.g., nightingale, Todt et al., 1979; e.g., indigo bunting *Passerina cyanea*, Rice and Thompson, 1968; Payne, 1996; e.g., marsh wren *Cistothorus palustris*, Kroodsma and Pickert, 1984; e.g., starling, Chaiken et al., 1994; e.g., zebra finch *Taenopygia guttata*, Slater, 1988; Eales, 1989; Mann et al., 1991; Chen et al., 2016). At the other extreme, some species require social interaction with a live tutor to develop normal song (e.g., sedge wren *Cistothorus stellaris*, Kroodsma and Verner, 1978; e.g., short-toed tree-creeper *Certhia brachydactyla*, Thielcke, 1984).

Songbirds do not arrive with a tabula rasa. Neural predisposition guides them to selectively learn and prefer conspecific models, and they may reject phrases of even closely related species (Marler and Peters, 1977), although social interaction can override a preference for conspecific songs (Gorton, 1977; Baptista and Gaunt, 1997). In some cases, birds will learn the songs of alien species with whom they have no social bond (Nottebohm, 1972; Baptista and Morton, 1981). For instance, songbirds that mimic (e.g., mockingbirds and lyrebirds) are not constrained by a species-specific template (Baylis, 1982). Despite their revelations concerning both instinct and learned behavior, Lorenz (1965) and other classical ethologists nonetheless held that the hard-wired “hereditary teaching machine which controls the primary programming” trumps learning and is central to understanding the process.

However, ethologists gradually moved away from drawing a hard line between “learned” and “innate,” the problematic labels of dichotimization that underpin nature/nurture (or nature/culture) debates (Johnston, 1988; Barlow, 1991). In place of categorical thinking’s strict border, biocultural labels like “inherited tendency” (Thorpe, 1958), “inborn blueprint” (Thorpe, 1961), “inherited or acquired auditory template” (Konishi, 1965); “instincts for inventiveness” (Marler, 1994), “template for learning preferences” (Marler, 1997), and “song templates” (Marler, 1997) mark attempts to account for the joint role of environmental *and* genetic instructions. With vocal learning guided and circumscribed by both in a complex intertwined process, contemporary research into the mechanisms of song learning continues to be a major project (Doupe and Kuhl, 1999; Adret, 2004; Bolhuis and Gahr, 2006). Even assigning a name and address to this “network of loops” distributed in multiple areas of the brain remains daunting (Reiner et al., 2004; Adret, 2008; Güntürkün, 2012). Terms like “natureculture” represent how scholars from other backgrounds, inspired by the work of Bateson (1980), also recognize the tendency for things inherited and acquired to percolate across the highly porous lines intended to confine them (Haraway, 2008; Fuentes, 2010; Taylor and Lestel, 2011; Latimer and Miele, 2013; Malone and Ovenden, 2017).

## Culture

Culture is often characterized as what is left in the container once genetic instructions are removed, but the topic is the site of frequent definitional contestation (Bohannan et al., 1973; Williams, 1976; Byrne et al., 2004; Hurn, 2012). Frustrating simple declarative sentences, culture is variously described as learned behavior (Mundinger, 1980) – or else learned behavior *and/or* information (Boyd and Richerson, 1985; Richerson and Boyd, 2005), depending on whom is canvassed. Both Rogers (1988) and Dean et al. (2014) emphasize the social learning inherent in the mechanism of cultural inheritance, while others suggest that culture and tradition are synonyms (Galef, 1992). So wide are the definitions of *culture* and the processes that propagate it that a study of it could be limited to humans (e.g., Kroeber and Kluckhorn, 1952; Tylor, 1968/1871; Sahlins, 1976) or expanded to take in more than 11,000 species (Lumsden and Wilson, 1981; Dean et al., 2014).

Eschewing one “true” definition, Byrne et al. (2004) promote a range of manifestations of culture in order to explore the richness of human and animal lives: culture as pattern, as sign of mind, as bonus, as inefficiency, as physical product, and as meaning. A variety of patterns of socialization may serve to transmit a given trait (Boyd and Richerson, 1985), and social information exchange may have interspecific bearing (Seyfarth and Cheney, 1990; Oda, 1998; Zuberbühler, 2000). Although culture may take in knowledge, values, skills, traditions, rules, thoughts, physical products, art, codes, social transactions, beliefs, and feelings, there is no reason to establish requirements that any one culture must accommodate *all* of these “key characteristics” (Laland and Janik, 2006). Neither should we allow anthropocentric, pretentious, or arbitrary stipulations that culture be classified as “sophisticated” or *Hochkultur* (e.g., Wallaschek, 1893; Dehaene, 2009). Cultural knowledge can be survival knowledge.

Laland (2017) details the dynamic of how culture transformed the evolution of human minds, arguing “Human minds are not just built *for* culture; they are built *by* culture.” Tomasello (1994) coined the metaphor “ratchet” to describe the accumulation over time of knowledge and iterative technological improvements. Cumulative culture rests on the development of traits that far exceed what one individual could invent alone and is often restricted to humans (Tennie et al., 2009; Dean et al., 2012, 2014). Cumulative culture and teaching reinforce one another and may have coevolved (Fogarty et al., 2011).

Although animals are seldom credited with ratcheting to the level of complexity found in cumulative culture, cultural transmission through imitation or instruction is nonetheless recognized in and integral to many species (e.g., Bohannan et al., 1973; Bonner, 1980; Mundinger, 1980; Cavalli-Sforza et al., 1982; Griffin, 1992; Stamp Dawkins, 1993; McGrew, 1998; Kitchener, 1999; de Waal, 2001; Rendell and Whitehead, 2001; Laland and Hoppitt, 2003; Whitehead et al., 2004; Laland and Janik, 2006; Sapolsky, 2006; Lycett et al., 2007; Laland, 2008; Bentley-Condit and Smith, 2010). Mesoudi et al. (2006) and Mesoudi (2011) suggest that cultural evolution shares fundamental features with biological evolution, while Ingold (2007) strongly pushes back against their account of human beings as “trait-bearing cultural clones whose only role in life is to express – in their behavior, artifacts, and organizations – information that has been transmitted to them from previous generations.”

Cultural transmission can occur via asocial learning, social learning, and teaching. *Asocial learning* describes the efforts of a single individual, such as learning by trial-and-error (Laland, 2017). It is widely accepted that animals also regularly exploit *social learning*, which allows an individual to rapidly acquire new skills or knowledge through observing and/or interacting with others. Ubiquitous in nature, social learning (or copying or imitation) occurs when an individual that is going about their business is copied without any active assistance to the learner. Some argue that a young bird learns in such a manner (benefiting inadvertently from public information), believing that instead of actively choosing to deliver targeted information, a song tutor is simply carrying out their quotidian tasks in the presence of an individual with less knowledge (Danchin et al., 2004). While a wealth of detail regarding songbird enculturation has been uncovered with a preoccupation on the apprentice, whether observational (social) learning can fully account for the cultural transmission of birdsong, or whether there could also be an element of active teaching involved, remains an open question.

## Teaching

Like culture, the subset of teaching is regularly claimed to be a uniquely human capacity, and literature on the subject is dominated by the human animal. As a vital psychological adaptation, teaching is ubiquitous in human societies (Boyd and Richerson, 1985; Fogarty et al., 2011; but see Paradise and Rogoff, 2009; Gaskins and Paradise, 2010). Teaching can hasten the acquisition of novel behavior (Boesch, 1991) and can solve adaptive problems that cannot be addressed by a learner alone (Kline, 2015). A review of current theoretical differences and contested definitions of teaching identifies three approaches to the topic: mentalistic, culture-based, and functional (Kline, 2015). *Mentalistic* descriptions put forward psychological and cognitive prerequisites. They hinge on mental state attribution (and an exaggerated sense of its importance) like theory of mind-based intentionality, foresight, and the ability to take another’s perspective (Boesch, 1991; Tomasello et al., 1993; Kruger and Tomasello, 1996; Boesch and Tomasello, 1998; Tomasello, 2000). In addition, they typically require the teacher to be attentive to shifts in a pupil’s competence (e.g., Barnett, 1968, 2008; Pearson, 1989; Strauss et al., 2002). Although teaching does not require language (Csibra, 2006; Gärdenfors and Högberg, 2015), linguocentrism and the *a priori* assumption that culture depends upon language constitute another hurdle for students of animal culture (e.g., Washburn and Benedict, 1979; Gracyk, 2013). However, others argue that human language is merely a special case of language and reject characterizing it as an exclusive capacity of a single species (Čadková, 2015). This harks back to von Uexküll (1934/2010), who (despite maintaining various exceptionalisms and hierarchies) made major contributions to theoretical biology: his concept of *Umwelten* describes how meaning is made everywhere all the time, thus disconnecting meaning from language’s tight grip. In addition, complex birdsong and other animal communication systems appear to transmit much more information than previously suspected and could contain language-like structure (Kershenbaum et al., 2014; Fishbein et al., 2020b). In fact, many species display sophisticated cognitive abilities that antedate human language (Fishbein et al., 2020a). Nonetheless, mentalistic approaches by default exclude animal teachers since intentions are notoriously challenging to infer in animals.

*Culture-based* models of teaching (advanced in cross-cultural psychology and sociocultural anthropology) replace the anthropocentrism of mentalistic approaches with Eurocentrism: teaching is what happens in formal Western classrooms (Kline, 2015). In the culture-based viewfinder, instances of social learning that might be described as informal, simple, observational, guided instruction, or practical learning do not qualify as teaching (e.g., Paradise and Rogoff, 2009; Gaskins and Paradise, 2010). This stance has a corollary in the arts, where the only legitimate cultural agent may be assumed to be the bourgeois Westerner. Here I side with Ingold (1993), who cautions against according “ontological primacy to the Western model of agency” rather than networks of multiple voices and relationships. While culture-based definitions require teachers in the foreground, other models argue for their place in the background. For instance, Vygotsky’s social constructivist classroom understands the learner to be accountable for their own learning, whilst the teacher is more a facilitator and co-collaborator than a didactic lecturer (Ozer, 2004). Other incursions upon culture-based definitions include flipped classrooms, which promote a participatory learning experience by replacing what was formerly teacher-led instruction with what was formerly self-directed homework (O’Flaherty and Phillips, 2015). Like mentalistic approaches, culture-based models are unproductive in animal research. By refusing to allow for a variety of transmission types even among humans, culturalist definitions seem to overlook the irony that “cultural learning is itself a product of culture” (Schneuwly, 1993).

*Functionalist* definitions understand teaching as behavior (or an array of behaviors) evolved to facilitate, expedite, or accelerate learning in others, but they reject a concentration on the teacher’s motivational state or on Western habits. With a focus on observable behaviors and teaching outcomes (Kline, 2015), functionalists inject biodiversity into the psychology of cultural learning. Proponents of this approach consider the potential adaptive benefits and fitness consequences of teaching behavior, as well as its evolutionary roots, and are open to the possibility that teaching is not an arena of human exceptionality (Ewer, 1969; Heyes and Galef, 1996; Fragaszy and Perry, 2003; Kline, 2015). In their seminal article, Caro and Hauser (1992) sought to move beyond narrow conventional definitions of teaching that cause species to go unstudied and instances to go unreported. Their straightforward functionalist definition encourages cross-species comparisons of behavior rather than attributions of mental states:

An individual actor **A** can be said to teach if it modifies its behavior only in the presence of a naïve observer, **B**, at some cost or at least without obtaining an immediate benefit for itself. **A’s** behavior thereby encourages or punishes **B’s** behavior, or provides **B** with experience, or sets an example of **B**. As a result, **B** acquires knowledge or learns a skill earlier in life or more rapidly or efficiently than it might otherwise do, or that it would not learn at all (Caro and Hauser, 1992).

Documentation of different forms of teaching in non-human animals has built on this definition and later tweaks, although refinements may risk “intentionality creep” if they impose additional cognitive criteria (e.g., Franks and Richardson, 2006; Thornton and McAuliffe, 2006, 2012; Richardson et al., 2007; Thornton et al., 2007; Hoppitt et al., 2008; Raihani and Ridley, 2008; Thornton, 2008; Thornton and Raihani, 2010). For instance, some have proposed that a bidirectional feedback loop between student and teacher is diagnostic of teaching, which would distinguish teaching from broadcasting (where an observer takes advantage of social learning without the assistance of a teacher) (Franks and Richardson, 2006; Richardson et al., 2007).

On the other hand, Byrne and Rapaport (2011) find the Caro and Hauser definition *overly* restrictive. They underline the value of an observational approach where instances of “most likely teaching” are treated provisionally as the real thing and not expunged out of fear of anthropomorphism (Byrne and Rapaport, 2011). Thinking in line with Laland and Hoppitt’s (2003) provocation, it seems fair to ask what proportion of human teaching could satisfy Caro and Hauser’s definition. With many anthropocentric assumptions under challenge at this historical moment, the question of the animal is the focus of spirited debate. The signal importance of Caro and Hauser’s model is that it seeks to decouple reports of teaching from mentalistic assessments in order to stimulate interest in this poorly understood area of animal behavior.

Scenarios and mechanisms suggesting animal teaching see a wide taxonomic distribution (in addition to the extensive review in Caro and Hauser, 1992, with sections on felids and other carnivores, on pinnipeds and cetaceans, on non-human primates, and on birds, also see von Frisch, 1967 on honeybees; Caro, 1995 on cheetahs; Guinet and Bouvier, 1995 on killer whale; Maestripieri, 1995, 1996 on non-human primates; Nicol and Pope, 1996 on domestic hens; Boran and Heimlich, 1999 on cetaceans; Rendell and Whitehead, 2001 on whales and dolphins; Roush and Snowdon, 2001 on cotton-top tamarins; Rapaport and Ruiz-Miranda, 2002 on golden lion tamarins; Krützen et al., 2005 on bottlenose dolphins; Franks and Richardson, 2006 on tandem-running ants; Radford and Ridley, 2006 on pied babblers; Thornton and McAuliffe, 2006 on meerkats; Raihani and Ridley, 2008 on pied babblers; Rapaport and Brown, 2008 on non-human primates; Bender et al., 2009 on atlantic spotted dolphins; Menzel et al., 2011 on honeybees; Bunkley and Barber, 2014 on pallid bats; Scheel et al., 2015 on chimpanzees; Wild et al., 2020 on dolphins). Of the numerous candidate cases scrutinized up to 1992 by Caro and Hauser, none as described exactly fit their definition. Certain issues could simply be unresolved technicalities, like reports of teaching that fail to state that the teacher does *not* modify their behavior in the presence of non-novices. Still, many of the other conditions in their definition are met (some are researcher’s observational data and not experimentally tested). Later scholarship uncovers strong cases that do meet their requirements, including tandem-running ants *Temnothorax albipennis* that guide naïve followers to a food source (Franks and Richardson, 2006), wild meerkats *Suricata suricatta* that teach their pups prey-handling skills (Thornton and McAuliffe, 2006), and an adult female pallid bat *Antrozous pallidus* that apparently assists a juvenile to learn a foraging task (Bunkley and Barber, 2014).

In addition to the caution built into the scientific method, other theoretical disputations moderate candidate cases, often cordoning off most or all areas of teaching as uniquely human (e.g., Tomasello et al., 1993; Premack and Premack, 1994, 1996; Povinelli and Preuss, 1995; Kruger and Tomasello, 1996; Bering, 2001; Strauss et al., 2002; Leadbeater et al., 2006; Csibra, 2006; Premack, 2007; Csibra and Gergely, 2009; Dehaene, 2009). Bracketing the animal with inability and the human with ability inhibits not just debate but field studies and seems unwarranted given recent theoretical and empirical developments in the area of animal cognition that reveal diverse intelligences (e.g., Reznikova, 2007; de Waal, 2009). Instead of characterizing teaching as an all-or-nothing phenomenon, we could allow for and expect distinct teaching mechanisms among species (e.g., Laland and Hoppitt, 2003; Hoppitt et al., 2008). This is consistent with Caro’s (2015) call for a comparative database of different types of teaching rather than focusing on “a single high-bar definition.”

The belief that songbirds are capable of not just learning but also teaching stretches back at least to Aristotle (Arbo and Arbo, 2006). DNA sequence data implicate Eastern Gondwana (Australia and Papua New Guinea) as the birthplace of songbirds (Edwards and Boles, 2002; Ericson et al., 2002; Norman et al., 2007). Many Australian birds are highly social (with long-term associations), cooperative, and long-lived, and their intelligence could exceed that of Northern Hemisphere temperate zone migrant species (Kaplan, 2015). (Male) songbirds that compete would likely differ in vocal behavior from males (and females) that cooperate (Kaplan, 2008). Nonetheless, with the notable exception of the Australian zebra finch (an opportunistic breeder with an unusually compressed developmental phase), studies, experiments, and theories have concentrated on the songs and vocal learning capacities of a handful of Northern Hemisphere species of male songbirds that compete (Brown and Farabaugh, 1997). Evidence is mounting that findings based on this subset are unrepresentative and lack comprehensive explanatory power, so considering the vocal behavior of a more representative Australian songbird stands to enhance current understanding of vocal learning and teaching. Below, I introduce the Australian pied butcherbird *Cracticus nigrogularis* into the wider debate surrounding mechanisms of cultural transmission and teaching.

## Ways and Means

### Study Species

The pied butcherbird is a member of the oscine family Artamidae. The two subspecies, *Cracticus nigrogularis nigrogularis* and *Cracticus nigrogularis picatus*, are indistinguishable in the field (Higgins et al., 2006). This sedentary mid-sized black and white songbird is distributed across much of mainland Australia (Higgins et al., 2006). Although the sexes are monomorphic, juvenile plumage is pale brown-gray for the first year. Social organization and behavior are poorly known (Higgins et al., 2006). Pied butcherbirds exhibit year-round territoriality, and one or more immature birds may remain to help feed and protect the next season’s nestlings (Robinson, 1994). While competition is the dominant model in birdsong neurobiology, a remarkably high proportion of Australian oscines are, like pied butcherbirds, cooperative breeders (Cockburn, 2003; Kaplan, 2008). While particularly common in Australia, cooperative breeding is rare in Northern Hemisphere songbirds (Cockburn, 2003). Helpers’ apparent altruistic behavior (postponing dispersal and reproduction, as well as taking on the costs of raising others’ young) poses an evolutionary quandary that was recognized by Darwin and continues to garner scientific interest (Darwin, 1859/1985; Wilson, 1975; Boland and Cockburn, 2002).

Pied butcherbird vocalizations are sonic heirlooms, the manifestation of likely millions of years of culture (Low, 2014). (While modifications to repertoire are enacted annually, it is not possible to claim this as cumulative culture since we have no recordings that span the substantial length of time necessary to speculate on this). Since 2005, I have spent 3–4 months annually listening, recording, and making observations on their vocal and other social behavior across the continent. Both sexes sing with formidable exuberance, including in duos and larger groups (Higgins et al., 2006; Taylor, 2008b). Duets and other diurnal group songs range from interchanges of apparently informal timing to intricate, coordinated performances, with the bulk of group song delivered in the hour after the dawn chorus. Material in group song may be repeated (exactly or with variation) several times before switching to new song phrases, usually with a regular change in songposts (Taylor, 2017). Brief interjections aside, only two types of solo singing practices are identified in pied butcherbirds: (1) *formal song* sees a soloist singing 1–3 s phrases of immediate variety and discontinuously, with a longer inter-phrase interval (often double the length of the phrase), and almost all of this is delivered nocturnally in the spring; while (2) *subsong* is delivered diurnally, where an individual might sing (usually softly) with immediate variety and almost non-stop for fifteen or more minutes, often incorporating elements of mimicry (Higgins et al., 2006; Taylor, 2017). The nocturnal solo songs of adult birds display strong individual variability (Janney et al., 2016; Taylor, 2017). Both solo and group song are noteworthy for their combinatorial complexity (additive process), but unlike most solo repertoire, group repertoire may see a strong overlap with neighbors and be stable over years (Taylor, 2017). Elements of group song may enter into solo repertoire (and perhaps vice versa).

In addition, pied butcherbirds excel at mimicry, including various avian species, other animals, and anthropogenic-sourced mechanical sounds (Higgins et al., 2006; Taylor, 2008a,b). Avian mimicry is poorly understood, with comparative studies suggesting that no single functional accounting can suffice for all mimicking species (Baylis, 1982; Dalziell et al., 2015). It is not known what motivates pied butcherbirds, with a good-sized repertoire of their own, to incorporate the sonic constructs of other species. Another Australian songbird, the lyrebird, learns their mimicry of alien species preferentially from other lyrebirds but may also learn from the original models (Powys et al., 2020); it is not known how the capacity for mimicry in pied butcherbirds might correlate to active teaching or vocal learning.

### Study Area

**FIGURE 1:**
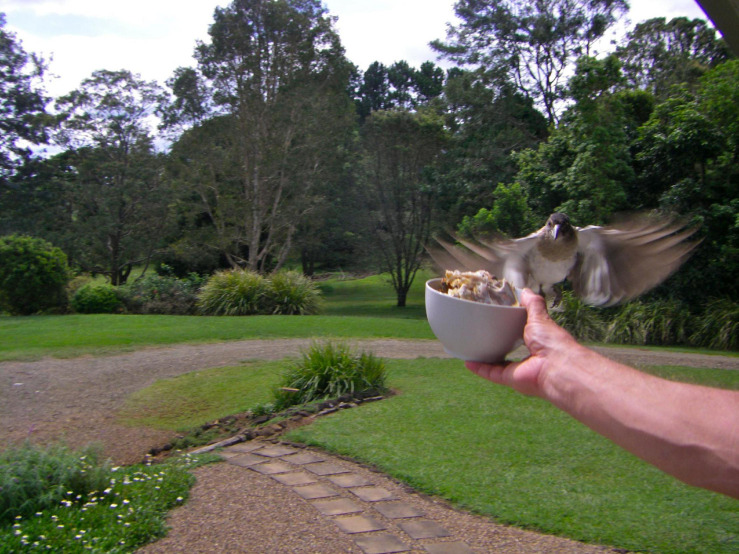
An immature pied butcherbird flying in to feed at the Maleny property.

**FIGURE 2:**
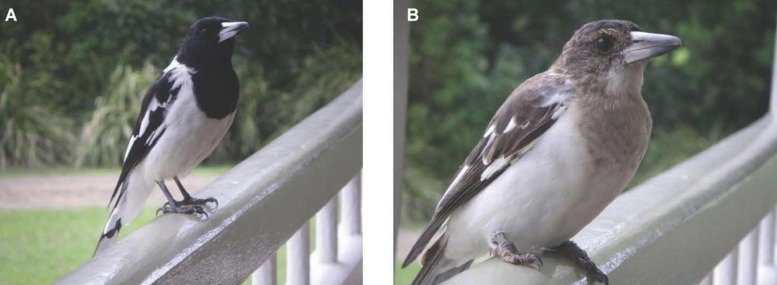
**(A,B)** An adult (l.) and an immature (r.) pied butcherbird perch on the deck rails, waiting for (and apparently singing for) a feeding at the Maleny property.

The study area is a 2.7-acre property in Maleny, Queensland, 56 miles north of Brisbane in the South Eastern Queensland Bioregion (GPS: 26°44′40.2″ S, 152°52′ 08.2″ E; 1,404 feet in elevation). I recorded nocturnal solo and diurnal group song at this property in the spring of 2008, 2009, 2010, 2012, and 2013, as well as diurnal group song in the autumn of 2008, 2010, and 2013. In this multispecies entanglement, free-living pied butcherbirds are habituated to the property owners, who feed and interact with them, and at times record their vocalizations (Figure 1). Since individuals are not banded, I base assumptions about the relatedness of the family on the straightforward field identification of immature birds (Figures 2A,B), the property owners’ accounts, and my own experiences at this property and hundreds of other field sites.

### Apparatus and Procedure

On 25 March 2013, I made sound recordings on an Olympus LS-10 Linear PCM Field Recorder with a pair of Sennheiser ME67 shotgun microphones mounted on a tripod. I took GPS measurements on a Garmin GPSmap 6.2s and photographs on a Pentax 8.l0 megapixels digital camera. I recorded behavioral observations at the end of each track, amplified by written annotations made on the same day. I assessed the recordings aurally and visually (in sonograms, which I generated with Raven Pro v1.6.1). Audio processing software included iZotope RX 7 Audio Editor for eliminating low bandwidth traffic.

This study takes place as part of longitudinal investigations into pied butcherbird vocalizations and concomitant behavior. Since I embrace the situated, embodied, and contested conditions of knowledge, fieldwork is fundamental to how I get my epistemological bearings (e.g., Haraway, 1988; Latour and Davis, 2015). In recognizing pied butcherbird agency, I eschew laboratory supervision and experimental control with the confidence that if my studies were dependent upon birds singing in such an impoverished setting, my understanding of their vocal world would be compromised. My project does not measure song development throughout a vocal ontogeny. Instead, my focus is on the spring nocturnal solo songs of these heretofore scarcely studied songbirds. I record and analyze their long solo songs (up to 7 h) in order to determine complexity, combinatorial rules, and other structural attributes, as well as their relationship to human music and musicality. With no reports of nocturnal solo song being delivered in the autumn, the rationale for these trips is to augment the vocal ethogram. Autumn trips typically yield bountiful group song, which could be significant in revealing the reach of a motif across territories, seasons, song types, and years.

## In the Field

Maleny, Queensland, Australia, 25 March 2013. At 5:00 AM, I set up the recording gear to a chorus of crickets and frogs and turn on the recorder at 5:10 AM. Supplementary Audio 1 documents entries to the dawn chorus arriving in this order: laughing kookaburra *Dacelo novaeguineae* (at 5:14 AM), pied currawong *Strepera graculina* (5:18 AM), pied butcherbird (5:28 AM), Australian magpie *Cracticus tibicen* (5:29 AM), yellow-throated miner *Manorina flavigula* (5:32 AM), mosquitoes (5:34 AM), and Eastern whipbird *Psophodes olivaceus* (5:38 AM). The pied butcherbird contribution comes from two immature and two adult birds, who deliver varied antiphonal phrases for 20 min beginning at 18:27 of the track (from 5:28 AM to 5:48 AM). Their vocal activity is then much reduced whilst the birds are feeding. Sunrise arrives at 5:54 AM.

**FIGURE 3:**
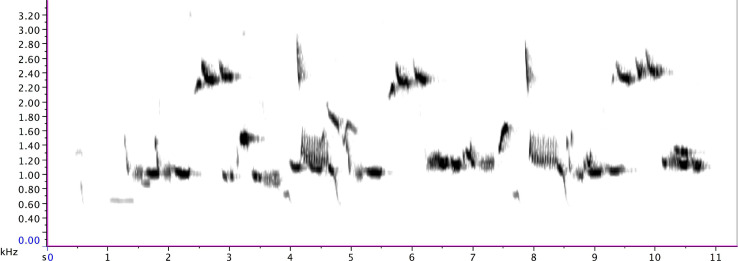
An extended group phrase sung by a pied butcherbird family at Maleny on 25 March 2013 (at 11:51 of Supplementary Audio 2).

**FIGURE 4:**
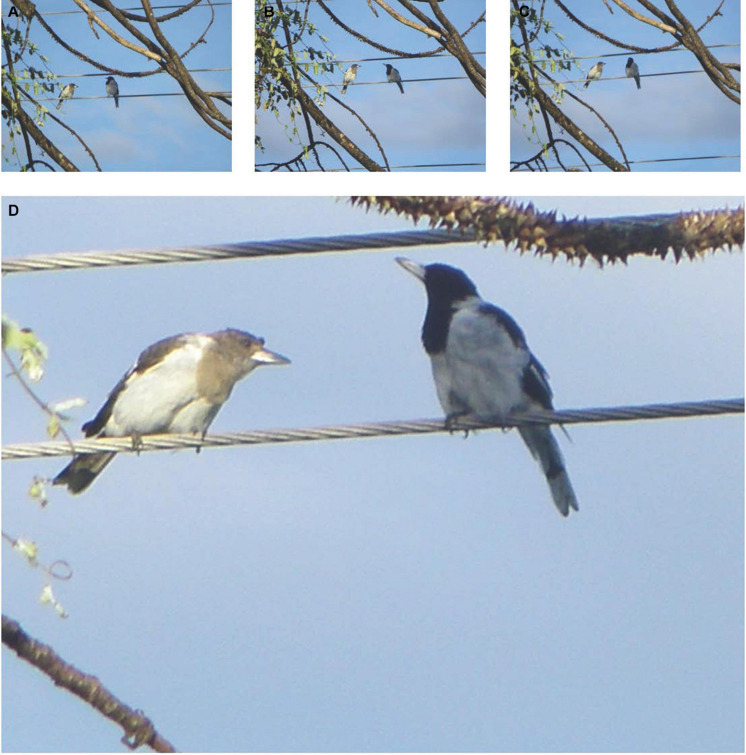
**(A–D)** An adult pied butcherbird sings whilst an immature bird faces the adult on 25 March 2013.

A new track (Supplementary Audio 2) commences at 6:17 AM, where this analysis will focus. Beginning at 0:15 in (the listed times below are track timings of Supplementary Audio 2 and not time of day), pied butcherbird vocalizations pick up again, with the delivery of 19 varied group phrases. As is typical, group singing is pieced together with motifs (a clearly defined subsection of a phrase) slotted into diverse combinations. Some group phrases run 10 s or longer (Figure 3). The pace of delivery fluctuates, and I expect the singing to be winding up by approximately 6:28 AM, based on my field observations on these and other individuals. However, at 12:59 in (6:30 AM), one individual recommences: an adult pied butcherbird perched high on a utility wire, facing an immature bird just inches away. The immature bird hunches over, looking toward the adult bird (Figure 4). Rather than singing with immediate variety, the adult sings quite repetitively, at times with a high rate of delivery, and with no conspecific vocal response. Singing for 25′59″ total, the adult raises and lowers their bill as the notes apparently demand and also delivers three “species calls” (a multi-purpose call deployed by the species) (Taylor, 2005).

**FIGURE 5:**
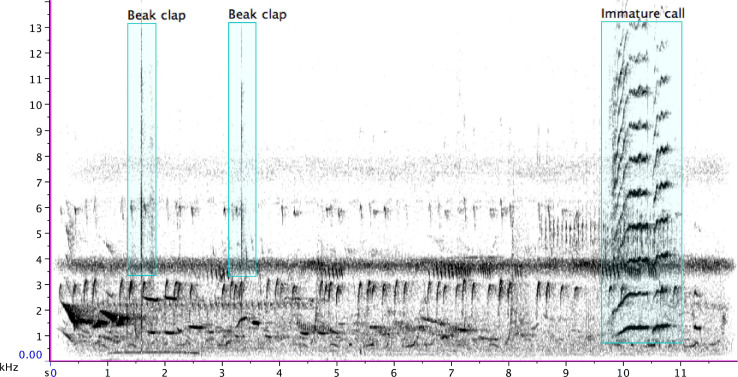
Beak claps of an adult pied butcherbird (1.3 s, 1.6 s, 3.3 s, and 9.3 s), followed by the “noisy” shriek of an immature bird (9.7 s), which includes harmonics that appear as stripes above the fundamental (at 18:29 of Supplementary Audio 2).

**FIGURE 6:**
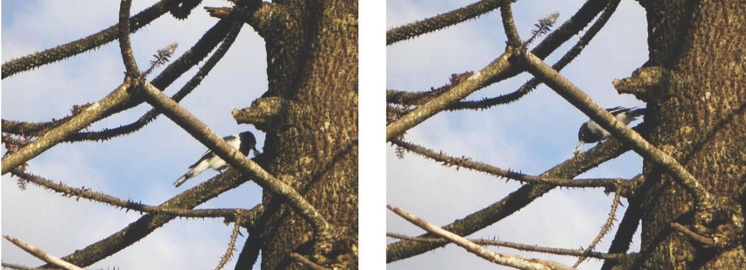
An adult pied butcherbird removes a morsel of cached chicken from their larder.

Twice, I hear a series of beak claps from the adult bird, which could be a threat display or the preface to (or sound of) a bite or stab. Following the first series of beak claps, the young bird flies off with a shriek (at 18:38) but immediately returns (Figure 5). At 38:59, the adult bird stops singing and flies down to their larder to retrieve a morsel of cached chicken (Figure 6). Holding it in their beak, the adult makes a soft barking sound that continues whilst the immature bird flies to the tree where the adult is, singing 8′58″ minutes total (from 40:08 to 49:06). A second immature bird also contributes a few phrases, which are softer or more distant from me, or both. In the end, the adult swallows or re-caches the treat and flies off. The track duration is 52′43″.

**FIGURE 7:**
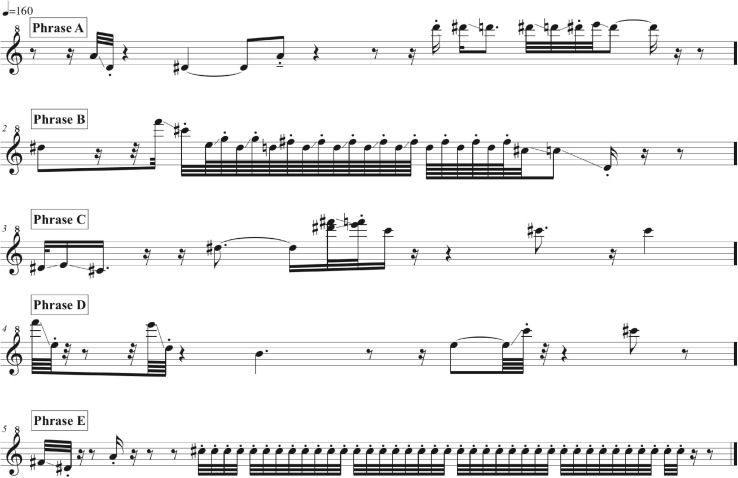
A music transcription of phrases A, B, C, D, and E as delivered by the adult bird as summarized in Supplementary Audio 3. ^#^Means sharp in music notation.

**FIGURE 8:**
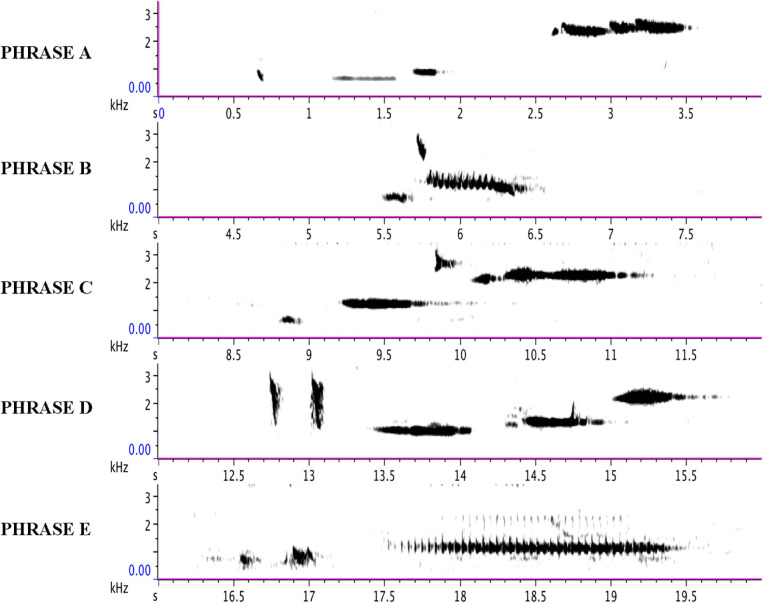
Each line of the sonogram displays one of the five phrases (A, B, C, D, and E) of the adult bird as delivered from 13:00 to 38:58 in Supplementary Audio 2 and summarized in Supplementary Audio 3.

Phrases A, B, C, D, and E as delivered by the adult bird are summarized in music notation in Figure 7, in a sonogram in Figure 8, in Table 1, and in Supplementary Audio 3, A distributional analysis (in this case, with letters assigned to phrases) of the adult bird’s phrases, with track timings of Supplementary Audio 2 beginning each line, reveals:

**TABLE 1:** A summary of the adult bird’s phrases, delivered in Supplementary Audio 2 from 12:59 to 38:58 = 25′59″ total.

**Phrase**	**Repetitions**	**Note morphology**	**Frequency range**
A	37	steep descending frequency sweep; low notes	569–896 Hz
B	9	ascending octave leap; descending frequency sweep; descending rattle (11 pulses in 0.5 sec., or 22 per sec.); low note	577–2,920 Hz
C	8	low note; double note	560–3,060 Hz
D	35	very steep descending frequency sweep making a “chip” sound; low note	991–2,791 Hz
E	5	low note; broad spectrum (“noisy”) rattle (40 pulses in 1.8 sec., or 22 per sec.)	577–1,421 Hz

12:59 A B A B B B A A A A A A A A A A A A A A A A A A A A A A A A

18:38 [beak claps followed by a shriek from the immature bird]

18:45 B A A A

19:33 [two species calls]

19:43 B A A A A A A A B B C C C C C C C C D D

24:52 [species call]

24:57 B A D D D D D D

28:36 [one bark]

28:37 D D D D D D D D D D D E E E E E D D D D D D D D D D D D D D D D

38:59 [barking begins in earnest].

The adult repertoire (with the following timings sourced from Figure 8 and Supplementary Audio 3) exploits a wide range of timbres and note types, including steep, swift frequency sweeps (e.g., 12.7 s and 13 s) at the beginning of Phrase D; a wide, ascending leap (5.5–5.7 s) in the first two notes of Phrase B; a descending rattle (5.8 s) in Phrase B; a broad spectrum (“noisy”) rattle (16.5–19.5 s) in Phrase E; an apparent double note (possibly sung from two sides of the syrinx) (9.85 s) in Phrase C; and notes at the low end of the species’ range (e.g., 0.7 s and 8.8 s) in Phrases A and C. Some phrases are delivered at a very high rate of repetition. For instance, Phrase A sees 37 iterations, with 27 delivered consecutively at a rate of almost 9 per minute (from 15:07 to 18:14).

**FIGURE 9:**
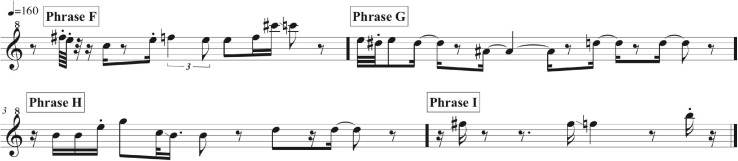
A music transcription of phrases F, G, H, and I as delivered by the immature bird as summarized in Supplementary Audio 4. ^#^Means sharp in music notation.

**FIGURE 10:**
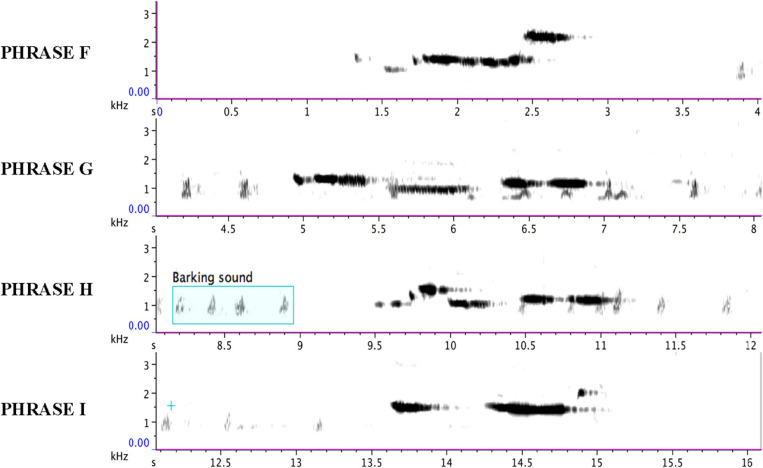
Each line of the sonogram displays one of the four phrases (F, G, H, and I) of the immature bird as delivered from 40:08 to 49:06 in Supplementary Audio 2 and summarized in Supplementary Audio 4. The regular soft “barking” sound of the adult bird is also visible on each line.

Phrases F, G, H, and I as delivered by the immature bird are summarized in music notation in Figure 9, in a sonogram in Figure 10, in Table 2, and Supplementary Audio 4. A distributional analysis of the immature bird’s phrases, with track timings of Supplementary Audio 2 beginning each line, reveals:

**TABLE 2:** A summary of the immature bird’s phrases, delivered in Supplementary Audio 2 from 40:08 to 49:06 = 8′58″ total.

**Phrase**	**Repetitions**	**Note morphology**	**Frequency range**
F	2	(only pure flute-like notes)	1,025–2,205 Hz
G	2	two notes warble slightly – a “quasi-rattle” whose units do not fully separate	913–1,361 Hz
H	10	(only pure flute-like notes)	999–1,533 Hz
I	11	(only pure flute-like notes)	1,387–1,998 Hz

40:08 F G H H H

40:49 [faster barking from adult bird]

41:01 H I F H H H I I I I I I I I I I G H H H.

None of the immature phrases match any of the material from the adult bird as sung from 13:00 to 38:58. Instead, the phrases are limited to relatively pure, flute-like notes (with the following timings sourced from Figure 10 and Supplementary Audio 4), although one immature phrase contains two notes that warble slightly (Phrase B from 5.1 to 6.1 s) – almost a “quasi-rattle,” whose units do not fully separate, as opposed to the adult bird’s well-separated rattles in Phrases B and E.

The frequency range for the adult bird is 542–3,023 Hz (C#^5^-F#^7^), spanning 2,481 Hz (two octaves plus a perfect fourth); for the immature bird, 913–2,205 Hz (A#^5^-C#^7^), spanning 1,292 Hz (one octave plus a minor third). Excluding calls, the delivery rate of song phrases for the adult bird is 94 in 25′59″, or approximately 3.6 per minute, and for the immature bird, 25 in 8′58″, or approximately 2.8 per minute. A comparative analysis of the 42 group songs delivered from 18:27 to 38:26 in Supplementary Audio 1 finds both adult and immature phrases (or parts of them) deployed as combinatorial components: phrase A (22x), B (13x), C (15x), F (4x), G (18x), and H (3x); some feature more than once in the same group song (summarized in Table 3). Phrase I does not feature in the group songs in Supplementary Audio 1. Visual contact was not sufficient to parse who (adult or juvenile) delivered which phrases.

**TABLE 3:** A tally of adult and immature phrases deployed as combinatorial components in group songs in Supplementary Audio 1 during the 20 min from 18:27 to 38:26 (each X = one delivery; some phrases feature more than once in the same group song).

**Timing**	**Phr A**	**Phr B**	**Phr C**	**Phr D**	**Phr E**	**Phr F**	**Phr G**	**Phr H**	**Phr I**
18:27									
18:31									
18:45								X	
19:05						X			
19:11						X			
22:12						X			
22:29					X	XX			
22:42						XX			
23:03						XXX			
23:44			XX			X			
25:40			XX				XX		
26:08			XX				XX		
26:58		X	XXX				XXX		
27:44			XX				XXX		
28:05	X	X							
28:19	X	X							
28:34	X								
29:00	X		X				XX		
29:28	XX						XX		
29:40	XX							X	
29:54	X								
30:05	X								
30:19		X							
30:26	X	X							
30:34	XX	XX							
31:01		X							
31:10	X								
31:20	XX							X	
31:30	X	X					X		
32:05	XX	X						XX	
32:43	X	X							
33:08	X								
33:21		X							
33:43			X						
34:25		X	XX				XXX		
35:07				X					
35:12									
35:18									
35:29				X					
36:22				X					
36:36				X					
38:24	X								
**TOTAL**	**22**	**13**	**15**	**4**	**1**	**11**	**18**	**5**	**0**

During the 52′43″ period of Supplementary Audio 2, neither the second adult pied butcherbird nor another human were seen or heard, nor was there any ambient noise of significance, either from the anthrophony (detritus like airplanes, vehicles, and other mechanical noises) or from the geophony (non-biological natural sound like wind, waves, and rain).

## At the Desk

Since birdsong is relevant to the study of both music and biology, it is worthwhile to consider what the workshop of a musicologist (or zoömusicologist) can contribute to our discussion. Musicology’s toolkit is well placed to move our discussion from the previous section’s description and transcription of this musical event to its upcoming analysis and interpretation. As a lifelong violinist/composer, I do not think of pied butcherbird song as music; I *hear* it as music – and not as an enchanting pastorale but as an intellectual exercise of close listening for me. Thoughts, arguments, and theories come later. Verdicts of human uniqueness shadow not just animal teaching but animal music, like the claim “informed listening requires the acquisition of language” (Gracyk, 2013). Because language may encode culture, some conflate the two. However, language, like music, is merely a subset of culture. Music and language do overlap in some parameters, with instances of resource sharing in neural processing (Patel, 2008), although Peretz (2006) and Peretz et al. (2015) argue that the overlap is minimal. Both consist of “culturally transmitted patterns whose specifics are biologically arbitrary yet obligatory in a given tradition” (Merker, 2005). Some projects offer linguistic-analytical explanations of music (Powers, 1980; Lerdahl and Jackendoff, 1983); others feel this has been less than fruitful (Lidov, 1997; Fink, 1999; also see Feld and Fox, 1994). There are also stark differences between the two. Lacking a fixed, unanimous reference, music elevates ambiguity to an asset, and rather than signaling a lack of imagination, repetition can serve as an essential tool. Higgins (2012) argues for music’s significance in the philosophy of mind as an alternative to models that frame the structure of language as the structure of thought. Music is a cognitive system. Rice (1997) makes a similar linkage, describing cases of “highly sophisticated non-verbal musical understanding” in practicing human musicians.

By analyzing avian sonic constructs as music, my work responds to Bateson’s (1972) provocation: “How is it that the art of one culture can have meaning or validity for critics raised in a different culture?” I build not only on heritage musicological tools and theory but also on work by ethnomusicologists (e.g., Nettl, 1983; Koskoff, 2014), New Musicologists (e.g., McClary, 1993; Kramer, 1995), anthropologists (e.g., Merriam, 1964; Feld, 1990), and sociologists (e.g., DeNora, 2000; Born, 2010), who have taught us to hear music from a much-expanded discursive space that recognizes music’s role in social formation. Human music, like birdsong, is more than its surface features; it is enacted in a social setting. Music is functional.

To my mind, placing birdsong solely within the domain of science endows the discipline with an unwarranted monopoly. Although my multidisciplinary birdsong research is robustly informed by the natural sciences and benefits from many of its knowledge claims, it does not walk in lock step with its practices of knowledge production. My grounding in musicology as a category of analysis prompts me to avoid using or inventing jargon for non-human animals that would duplicate universal terms developed over the centuries to describe and analyze sound. Instead, applying human music’s terminology to birdsong is central to my work, although claiming blanket equivalence is not.

We are now better equipped to return to our Maleny vocalists. Below, I test the event against Caro and Hauser’s guidelines.

*A teacher must modify its behavior only in the presence of a naïve observer, and this must come at some cost or at minimum without immediate benefit to the teacher.* Active teaching is perhaps the most complex form of sharing knowledge amongst non-human animals (Reznikova, 2007), and as such is a costly path for a song tutor (Merker, 2005; Merker and Okanoya, 2007). Caro and Hauser (1992) make the further point that cases of purported teaching that most intrigue researchers are behaviors that conspicuously deviate from a species’ customary repertoire, rather than being merely rare occurrences. Kline (2015) also underlines the importance of distinguishing between actors’ baseline behaviors and those during a teaching episode, since this might assist in clarifying the stipulation *only in the presence of a naïve observer.* The Maleny adult’s behavior marks a major modification since it fits neither of the documented song types, formal song or subsong. It combines elements of formal nocturnal song (discontinuous singing and an inter-phrase interval – but not nocturnal, not in the spring, and not sung with immediate variety) with elements of subsong (diurnal delivery – but not soft, not non-stop, and without mimicry) (Table 4). Crucially, in neither formal song nor subsong is the singing with eventual variety, as it is in the Maleny event. This is the sole example I can point to in my catalog where a pied butcherbird sang with eventual variety, as well as the sole example where a bird sang solo phrases with another bird facing them and in such close proximity (*duetting* birds will sing from the same tree). Taken together, the teacher’s highly repetitive singing at a later time of day than normal (which matches no known or documented pied butcherbird song type), with no answer from a conspecific, and while facing a naïve bird from just inches away – all are extraordinary. All vocal signaling comes at a cost: singing takes time from potentially more imperative activities like predator vigilance and foraging (Oberweger and Goller, 2001; Merker, 2012). The Maleny adult modified their behavior at some cost (setting a high-fidelity example and singing for 25′59″) without apparent immediate benefit. However, I am unable to rule out that this individual *never* behaved like this in the presence of another adult bird. The proviso seems not just a high bar but an impossible one. Even if my educated hunch is correct about this event, as a matter of logic, the lack of an ability or the absence of a cause or action cannot be demonstrated (Hauser, 1993; Laland and Hoppitt, 2003). I recommend removing “only” from this part of the definition.

**TABLE 4:** A comparison of species-typical song characteristics in pied butcherbird formal song, the Maleny song event, and subsong.

**Song characteristics**	**Formal song**	**Maleny song**	**Subsong**
Discontinuous singing (regular inter-phrase interval)	X	X	
Continuous singing (no inter-phrase interval)			X
Singing with immediate variety	X		X
Singing with eventual variety		X	
Delivered nocturnally	X		
Delivered diurnally		X	X
Delivered only in the spring	X		
Delivered beyond the spring		X	X
Delivered with a strong signal	X	X	
Delivered softly in less than full voice			X

*The teacher’s actions must encourage or punish the pupil’s behavior, or provide the pupil with experience, or set an example.* Social influences impinge upon a young bird engaged in vocal learning. In this case, the teacher has played an active role, apparently both punishing (beak clap threats and possibly a peck, judging from the shriek) and encouraging (tempting with a morsel from the larder) the pupil. Such behavior could take in nuances like “approval” and “disapproval,” “feedback” and “bribe.” My previous encounters with the barking sound at this and other locations suggest it is deployed when in competition for food (Taylor, 2017), although I find nothing in the literature detailing an adult bird offering a food reward to a vocal student. Also relevant is that human listeners readily hear pied butcherbird songs as musical, and those who feed meat scraps to their local birds report that that they “sing for their supper” or reward humans with song, although this has not been formally studied. I have witnessed numerous such cases, including both the teacher and pupil in question. If these birds do “sing for their supper” to humans, can we allow for the possibility that they might translate the behavior to their own kin? As for the pupil, the brief flight away, the shriek, the return to the utility line close to and facing the adult, the singing for 8′58″ at the end – these all could suggest reactions to *encouragement and punishment*.

Beyond carrot-and-stick approaches, this section of the definition can also be met by *providing the pupil with experience* or by *setting an example*. The long period of time that the adult held the morsel in their beak and delivered the barking sound could be interpreted as provisioning an experience, since when the immature bird began to sing, the adult might have stopped immediately but instead persisted. In addition, in a focus on sound, there may be an assumption that only one modality is open. Nonetheless, extra-auditory influences act at and value add to multiple levels of song development (West and King, 1996). In an article on demonstration and pantomime in teaching, Gärdenfors (2017) makes the case that “showing how to do” is a crucial step separating animals and humans, since it requires advanced mind-reading. Although the communicative function is not known, a singing pied butcherbirds’ multimodal display sees them alternate a standard upright posture with raising the bill high (often for higher-pitched notes) and sinking it on the breast (for lower-pitched notes) – which could be a product of physical or physiological constraints rather than (or as well as) part of a coordinated visual display – along with opening or even flapping wings (Taylor, 2017). The Maleny adult bird’s whole-body motor performance is consistent with this. Birdsong is a multifaceted communicative device designed to draw and focus attention through the manipulation of sound and movement. Could it be that part of the lesson was a close-up demonstration of body comportment when vocalizing high and low notes, rattles, steep frequency sweeps, and other technical challenges? (When pied butcherbirds sing solo or together, they are usually more physically distant from one another; in addition, this would mark the only occasion I witnessed two individuals facing each other whilst one is singing.) Harking back to the mirror neurons that mediate song in oscines, it seems fair to ponder if this demonstration provoked kinesthetic empathy in the naïve viewer, where what is perceived is linked to how to perform it and the energy costs involved. All four gradations of this requirement appear to be met.

*As a result, the pupil must acquire knowledge or learn a skill earlier in life or more rapidly or more efficiently than they might otherwise do, or that they would not learn at all.* Skills and information that are difficult or impossible to acquire in the absence of teaching are powerful pointers to the presence of teaching (Ewer, 1969; Ridley and Ashton, 2015). Nowhere is deliberate teaching more essential than in “the acquisition of complex arbitrary patterns” (Merker, 2005). The melodic, timbral, rhythmic, and combinatorial diversity of pied butcherbird vocalizations keep company with the most extravagant and challenging in the avian world. Not all human musical capacities depend on formal musical training (Bigand and Poulin-Charronnat, 2006; Peretz, 2006). Nonetheless, I imagine that a human music student presented with this level of difficulty (Figures 7, 8) would be greatly stimulated and aided by guidance from a teacher, so perhaps a pied butcherbird would as well. In addition to a teacher’s advocacy, there is the utility song provides to both teacher and learner. Singing is a family affair. Brown and Farabaugh (1997) argue that vocal learning is targeted and local, allowing a songbird to share vocalizations with a particular subset of conspecifics. Further, teaching is believed most common when costs to teachers are low and benefits to students high (Galef et al., 2005; Thornton and Raihani, 2008). An immature pied butcherbird would be expected to join in cooperative territorial defense, with potential fitness consequences for the family group. I would prefer to linger with the purported teacher, who has provided a repetitive, focused singing demonstration that directly assists the pupil in learning challenging repertoire more rapidly and efficiently than they might otherwise do. However, the words *As a result, the pupil must acquire* demand a response. Whilst the adult bird’s actions seem to tick the box, producing proof that the pupil has acquired this knowledge or skill is problematical. Given the learning proclivities of immature songbirds, it is possible that the Maleny event was one of only a handful of apparent singing lessons – and since pied butcherbirds are known to be vocal learners, that aspect is not in question. Perhaps a bit of anthropocentrism has crept into this part of the definition – for songbirds, there will be no call-and-response lesson. Despite studies of memory mechanisms showing birds like nightingales can learn with very limited exposure (Hultsch and Todt, 1989a), vocal production lag time conflicts with the problematical requirement central to much teaching theory that there be no period of delay between instruction and production, an issue Caro and Hauser (1992) acknowledged as unresolved. Measuring what was learned based on vocal production may underestimate what was memorized but not (yet) produced in a pupil, so delays in benefit may be expected for *both* student and teacher. With oscines so significantly underwriting our knowledge of the capacity for vocal learning, prerequisites that virtually exclude them from teaching are highly problematical.

Despite the episode failing to meet this aspect of the definition, I want to stay momentarily with the issue of relatedness, a recurring theme in teaching since it is considered an altruistic act (Caro and Hauser, 1992; Galef et al., 2005; Tennie et al., 2009). In my experience, the adult pied butcherbird at this time of day (6:30 AM) and at this season (autumn) would not be singing solo phrases at all – or at most, they might deliver several phrases in this 25′59″ time period. Instead, they would be feeding. Could this be an example of altruism? Teaching is thought more prevalent when the teacher and student are closely related (Galef et al., 2005; Thornton and Raihani, 2008). Fogarty et al. (2011) argue that cooperative breeders like ants, bees, meerkats, and pied babblers provide the most compelling instances of animal teaching, possibly on account of shared provisioning costs, resulting in a lower per capita cost—and I again note that pied butcherbirds are cooperative breeders, although no helpers-at-the-nest were observed in this year by the author or the property owners. Laland (2017) questioned this odd assortment of animals, arriving at a similar conclusion: “What cases of animal teaching have in common are a high degree of relatedness between tutor and pupil, factors that reduce the costs of teaching, and an otherwise difficult-to-learn skill that confers a substantial fitness benefit.” Tolerance of close observation also seems applicable here, with these birds engaged in intense joint attention with one another. Their interaction would not be an isolated event but instead would be situated within a wider context of sociality and reciprocal relationship.

Finally, more stringent definitions require the teacher to be *sensitive to the student’s changing competence and modify their behavior to these changing skills* (Premack and Premack, 1994). Barnett (1968) insists that the teacher must persist, perhaps even adapt, until the student achieves an acceptable outcome. The Maleny tutor provisioned five motifs/phrases that the immature bird had apparently not yet learned or perhaps not yet attempted to sing. While a mentalistic add-on requirement is not essential to make my case, the level of difficulty of the adult song as heard by the ear and represented in music notation and sonographic analysis stands in striking contrast to the much simpler repertoire of the immature bird. Since pied butcherbirds apparently lack a human language counterpart with which to enhance a lesson plan and impart complex subject matter, they must find another way through. Is this a case of evaluative teaching, with the lesson adjusted to the level of skill attained by the immature bird? One could speculate that the recording, music transcription, and sonogram serve as a behavioral readout of an internal state – that the adult recognizes that the apprentice lacks a certain skill and has targeted the deficiency. However, if our definition insists that a non-linguistic agent confirm in some other manner that they have chosen developmentally appropriate phrases, this aspect of the definition remains inconclusive and unproven.

This episode underscores the need for a definition of, or a range of characterizations of, teaching that link explanations of how songbirds learn to explanations of how songbirds (might) teach. The striking neural, cognitive, and molecular parallels between vocal learning in birds and human language acquisition suggest not just comparable morphological adaptations but also comparable behavioral ones (Bolhuis et al., 2010). We cannot foreclose the possibility that these parallels extend to the closely related activity of teaching.

## Whose Evidence?

We began by noting that despite the considerable amount of ink spilt on vocal learning in songbirds, the topic of teaching is all but neglected. In the account given above, quantitative engagements and qualitative narratives guided by an expert in the species converge to suggest that a cultural apprenticeship may have been supported through active teaching. However, some components of the Caro and Hauser definition remain unfulfilled, even as the episode appears to exceed other more stringent requirements. Moreover, the adult and immature birds’ actions have been seen, heard, and measured – but not replicated. Neither is this study tied to individuals’ full life history or based on banded birds and empirical testing. On the other hand, while variables are largely controlled out of laboratory studies, an artificial environment lacks the rich circumstances a young bird requires to develop normally (Kroodsma, 2004). Just as Northern hemisphere songbirds cannot supply a complete description of oscine behavior, neither can laboratory results seamlessly reflect what actually occurs in the field (Kroodsma, 1982, 1996; Clayton, 1989; Beecher, 1996). Although laboratory and field observations can be complementary, “field behavior has epistemic primacy” (Clayton, 1989). Additionally, a wealth of precision measuring and recording devices can accompany researchers into the field.

Thus, when instances of purported animal teaching are reported, the question “whose evidence?” is germane. In asking how far observational evidence can take us, a number of ethologists have argued that careful anecdote and other qualitative reportage of rarities and one-offs, of the fleeting and the subjective, merit wider consideration. These come into their own when crafted by those who have cultivated “a feeling for the organism” (Fox Keller, 1983) – observers attuned to the nuances of an animal’s communication system and proficient in careful structural descriptions of behavior (Wemelsfelder et al., 2001; Whiten and Byrne, 2010; Kaplan, 2015). Bekoff’s (2000) claim that “the plural of anecdote is data” highlights the confidence that ensues as multiple records are reported and collated and the comparative framework expanded (Whiten and Byrne, 2010). This is consistent with Darwin, whose unit of selection was the individual (Crist, 1996). Experts’ observational data are precious evidence for studying anomalies unavailable to laboratory-bound researchers.

Similarly, Despret (2013) roundly champions the subjective experiences of scientists with “risky practices”: “Rather than being unscientific, empathy becomes a scientific tool, a tool that needs to be shaped, forged, refined, and embodied.” Daston and Mitman (2005) have documented how the tendency of ethologists to anthropomorphize increases, not decreases, as their experience with a species accumulates. Meanwhile, Prum (2017) has pushed for an evolutionary theory that embraces *non-human animals’* subjective experiences, since these give rise to “critical and decisive consequences for their evolution.” Might the threat anecdote presents to the validity of the scientific method (used to explore research questions guided by a dislocated, dispassionate observer) be waning? In my work, critically assessed input from everyday people, citizen scientists, and others (who may lack formal credentials but nonetheless possess significant knowledge) provides a valuable supplement. Likewise, the natural sciences have begun to recognize the potential of ordinary people to increase animal observations on both time and geographic scales (e.g., Cohn, 2008; Bonney et al., 2009; Hecht and Cooper, 2014). Citizen scientists and other perspectives that we might only source from anecdotes stand to document rare and novel behavior previously disregarded in knowledge enterprises. In order to tell the fullest story possible about animal teaching, the window must be opened wide.

In part, the dearth of evidence for teaching in non-human animals may reflect the difficulty in framing evidence and mustering unequivocal support for it rather than its absence (Thornton and McAuliffe, 2006). Since disciplinary caution may prompt biologists to avoid “teaching/teacher” altogether, it is not unusual to come across research describing how a dolphin is a “demonstrator” (Wild et al., 2020), while a science journalist popularizing the same report straightforwardly interprets the study as an account of dolphins who “teach” and dolphin “teaching” (Langley, 2020). Moreover, Latour has called attention to the constructed nature of documentation in science culture, asking “are data a subset of narratives, or an opposition to narratives, or are narratives inside data?” (Latour and Davis, 2015). Scientists must constantly decide which data to retain and which to ignore, and accounts of intriguing animal behavior situated outside a scientist’s methodology or testable hypothesis may be branded “officially unusable” (Dennett, 1987). In these days of dwindling funding along with pressure to publish (quickly) or perish, the rarity of reports on (and possibly the rarity of occurrence of) animal teaching makes the subject a precarious research enterprise. We can add to this the tyranny of journal impact factor that encourages theory over field studies. While broader theoretical discussions that set field studies in context and search for their meaning are laudable, as Tinbergen (1963) observed, “Contempt for simple observation is a lethal trait in any science.” It could be that reports of animal teachers will arrive principally from opportunistic observations made whilst conducting other research rather than in controlled laboratory settings. Many of the scholars I have cited believe they can maintain rigor whilst drawing on observational evidence. Bekoff (2000) urges that the burden of proof be shared – that skeptics actively defend their position, rather than hiding behind “we can never really know.”

My advocacy for a broad, inclusive appreciation of teaching, rather than rehearsing definitions that refine and narrow the activity, builds on ethologists who have appealed for a moratorium on claims of discontinuity, with humans as the center of meaning, knowing, and value (Scheel et al., 2015; de Waal, 2016). In theory, scholars have a right to craft a restrictive definition based on the ontological difference inherent in their belief system, even if it is overly generous to ourselves or constructed without taking animals into account. For example, Barnett (2008) bemoans the neglect of teaching as a subject of study, although in the same sentence declares it “distinctively human.” However, how such borders are drawn and policed piques my interest when the politics of dividing the human and non-human becomes a barrier to research. Categorical rejection of animal teaching is an affront to the open-mindedness upon which scientific discovery is founded.

With so few species canvassed to this point, our current over-reliance on an extreme case – the psychological and mentalistic mechanisms of human culture – is highly premature. In their avoidance of a monolithic view of teaching, Thornton and McAuliffe (2012) would widen our understanding of skill monitoring, believing that “responses to physical or behavioral cues may suffice for teaching to be targeted appropriately.” The acceptance of minimal criteria will encourage further investigations and reports. Granted, in even the most thoughtful and generous characterization, something of promise will be left out, whilst something else of dubious merit will find its way in. Nonetheless, since categories at their edges are where we find noteworthy goings-on, if enough reports on the cluster of practices linked to the term “teaching” across a wide and uneven range of taxa are cataloged and systematically compared, the field will progress beyond the “most likely teaching” versus “we can never really know” impasse. In this, songbirds’ well-documented capacity for vocal learning places them in a potentially key strategic position in the related study of teaching.

## Data Availability Statement

The original contributions presented in the study are included in the article/Supplementary Material, further inquiries can be directed to the corresponding author.

## Ethics Statement

Ethical review and approval was not required for the animal study because the study relies on opportunistically recording birds and the soundscape. The Macquarie University Animal Ethics Committee reviewed the research project and determined that no ethics approval was required.

## Author Contributions

HT wrote, edited, and approved the manuscript.

## Conflict of Interest

The author declares that the research was conducted in the absence of any commercial or financial relationships that could be construed as a potential conflict of interest.
